# Theoretical Investigation into a Possibility of Formation of Propylene Oxide Homochirality in Space

**DOI:** 10.1089/ast.2022.0005

**Published:** 2022-10-31

**Authors:** Yuta Hori, Honami Nakamura, Takahide Sakawa, Natsuki Watanabe, Megumi Kayanuma, Mitsuo Shoji, Masayuki Umemura, Yasuteru Shigeta

**Affiliations:** ^1^ Center for Computational Sciences, University of Tsukuba, Ibaraki, Japan.; ^2^ Research Center for Computational Design of Advanced Functional Materials, National Institute of Advanced Industrial Science and Technology, Tsukuba, Ibaraki, Japan.

**Keywords:** Propylene oxide, Circularly polarized light, Homochirality, Lyman-αregion, Density functional theory

## Abstract

The preferential synthesis or destruction of a single enantiomer by ultraviolet circularly polarized light (UV-CPL) has been proposed as a possible triggering mechanism for the extraterrestrial origin of homochirality. Herein, we investigate the photoabsorption property of propylene oxide (*c*-C_3_H_6_O) for UV-CPL in the Lyman-α region. Our calculations show that *c*-C_3_H_6_O was produced by CH_3_^+^ and CH_3_CH(OH)CH_3_ or C_3_H_7_^•^ and O (triplet). The computed electronic circular dichroism spectra show that *c*-C_3_H_6_O and the intermediate (CH_3_CH(OH)CH_2_^+^) could absorb the UV-CPL originating from the Lyman-α emitter spectrum, suggesting that the photolysis of *c*-C_3_H_6_O or CH_3_CH(OH)CH_2_^+^ upon irradiation could induce chiral symmetry breakage.

## 1. Introduction

Chiral organic molecules cannot be brought into congruence by translation, rotation, or conformational changes, while they are distinguished by two mirror images. The enantiomers of chiral molecules have two absolute configurations denoted as *R* and *S* (l and d in biomolecules). Most organisms on Earth selectively use l-form amino acids and d-form sugars for their body compositions, and this natural selection is called homochirality. The origin of homochirality has been a key mystery in the study of the origin of life on Earth and has long been a matter of controversy.

Since the discovery of the enantiomeric excess (*ee*) of amino acids, including that in the Murchison meteorite (Cronin and Pizzarello, 1997; Engel and Macko, 1997), the cosmic origin of homochirality has been the focus of several studies. Various enantiomer formation and amplification mechanisms have been suggested and discussed (Kondepudi *et al.,* 1990; Bonner, 1991; Soai *et al.,* 1995; Cronin and Pizzarello, 1997; Engel and Macko, 1997; Bailey, 2000; Kawasaki *et al.,* 2006; Fletcher *et al.,* 2007; Garcia *et al.,* 2019; Glavin *et al.,* 2020). One of these mechanisms is the preferential synthesis or destruction of a single enantiomer through exposure to ultraviolet circularly polarized light (UV-CPL). This mechanism has been proposed as a possible triggering mechanism to induce asymmetry in amino acids (Bailey *et al.,* 1998; Garcia *et al.,* 2019; Glavin *et al.,* 2020) as a physical process, which is widely recognized as one of the extraterrestrial origins of homochirality. Indeed, the selective destruction of enantiomers by UV-CPL has been confirmed in laboratory experiments (Flores *et al.,* 1977; Meierhenrich *et al.,* 2005, 2010; Nuevo *et al.,* 2007; Meinert *et al.,* 2014, 2015; Tia *et al.,* 2014). In addition, infrared CPL of up to 17% was detected within the high-mass star-forming regions of the Orion molecular cloud (OMC-1) (Bailey *et al.,* 1998). The infrared CP image shows that the infrared CPL region was spatially extended around young stellar objects (Fukue *et al.,* 2010; Kwon *et al.,* 2013, 2014, 2016, 2018), which were significantly larger than the size of our solar system. Recently, high infrared circular polarization induced by scatterings from dust grains aligned in magnetic fields has been explored by radiative transfer calculations (Fukushima *et al.,* 2020). Such experiments and measurements are consistent with the astrophysical scenario of the origin of homochirality. In particular, in the early phase of the galactic evolution, the strongest emission in the pan-galactic light is the Lyman-α (Lyα) line with an emission of 10.2 eV (121.6 nm). This emission is caused by relaxation from the first electronic excited state to the ground state of a hydrogen atom. Such galaxies emitting Lyα radiation are observed as Lyman-α emitters (LAEs) (Shibuya *et al.,* 2014). Therefore, the consideration of the photolysis of chiral molecules by Lyα irradiation can provide insights into a possible triggering mechanism to induce asymmetry in amino acids.

McGuire *et al.* (2016) detected a chiral molecule—propylene oxide (*c*-C_3_H_6_O)—for the first time in the Sagittarius B2 star-forming region using a telescope. However, the possibility of the existence of *ee* in the case of *c*-C_3_H_6_O could not be determined. Nevertheless, knowing the possibility of its existence is of significant importance to deeply understand the origin of homochirality. Considering the astrophysical scenario of the destruction of enantiomers through CPL irradiation, herein, we investigated the possibility of *ee* generation for *c*-C_3_H_6_O using quantum chemical calculations. We determined the formation pathways of *c*-C_3_H_6_O based on those of ethylene oxide (*c*-C_2_H_4_O) (Dickens *et al.,* 1997; Turner and Apponi, 2001; Bennett *et al.,* 2005). Furthermore, we calculated the oscillator and rotational strengths of electronic excitation for the chiral species during the formation processes to discuss the possibility of photolysis by UV-CPL absorption in the Lyα region.

## 2. Methods

### 2.1. Computational details

All calculations were performed by using the density functional theory (DFT) and post-Hartree-Fock (post-HF) methods implemented in the Gaussian 16 program package (Frisch *et al.,* 2016). We performed full geometry optimizations using DFT calculations with the B3LYP functional and the aug-cc-pVTZ basis set, and then determined the energies using post-HF calculations with the CCSD(T) level of theory and the aug-cc-pVTZ basis set for the optimized geometries. Our previous DFT calculations confirmed that the B3LYP functional suitably reproduced the geometrical structures and total energies, which were in close agreement with the reliable CCSD(T) results (Kayanuma *et al.,* 2017; Sato *et al.,* 2018; Shoji *et al.,* 2022). By calculating the analytical harmonic vibrational frequencies, we confirmed that the obtained local minima and transition states have no and one imaginary frequency mode, respectively.

Electronic excitation energies, oscillator strengths, and rotational strengths were calculated for the chiral species during the synthesis of *c*-C_3_H_6_O by using time-dependent density functional theory (TD)-DFT calculations with the CAM-B3LYP functional and the daug-cc-pVQZ basis set. We calculated 100 excited states to obtain the electronic circular dichroism (CD) spectra, in which Gaussian functions with a bandwidth of 0.1 eV for each excitation position (Rizzo and Vahtras, 2011) were used in constructing the spectra.

### 2.2. Selection of reaction pathways

We focused on the formation of ethylene oxide (*c*-C_2_H_4_O) to investigate the reaction pathway of *c*-C_3_H_6_O formation. In previous studies, some *c*-C_2_H_4_O formation pathways, based on the interstellar environment in hot-core and star-forming regions such as Sagittarius B2(N), have been proposed (Dickens *et al.,* 1997; Turner and Apponi, 2001; Bennett *et al.,* 2005). The three types of proposed formation pathways are as follows.

(1.1) $$C{H_{3}}^{+}+C_{2}H_{5}OH\to C_{2}H_{5}O^{+}+CH_{4}$$

(1.2) $$C_{2}H_{5}O^{+}+e^{-}\to c-C_{2}H_{4}O+H^{∙}$$

(Dickens *et al.,* 1997),

(2) $$O+C_{2}{H_{5}}^{∙}\to c-C_{2}H_{4}O+H^{∙}$$

(Turner and Apponi, 2001), and

(3) $$C{H_{3}}^{+}+HCHO\to c-C_{2}H_{4}O+CH_{2}CHOH$$

(Bennett *et al.,* 2005). No details on path 3 are available in the literature.

Based on the above pathways, the following three types of *c*-C_3_H_6_O formation pathways were investigated:

(A−1) $$C{H_{3}}^{+}+CH_{3}CH\left(OH\right)CH_{3}\to C_{3}H_{7}O^{+}+CH_{4}$$

(A−2) $$C_{3}H_{7}O^{+}+e^{-}\to c-C_{3}H_{6}O+H^{∙}$$

denoted as path **A**,

(B) $$O+C_{3}{H_{7}}^{∙}\to c-C_{3}H_{6}O+H^{∙}$$

denoted as path **B**, and

(C−1) $$C{H_{3}}^{+}+CH_{3}CHO\to CH_{3}OCHC{H_{3}}^{+}$$

(C−2) $$CH_{3}OCHC{H_{3}}^{+}+e^{-}\to c-C_{3}H_{6}O+H^{∙}$$

denoted as path **C**. Path **C** was considered by using only the reactant species and stoichiometry of path 3 because the details have not been mentioned.

## 3. Results and Analysis

### 3.1. Reaction pathways

#### Path **A**

**FIG. 1.**
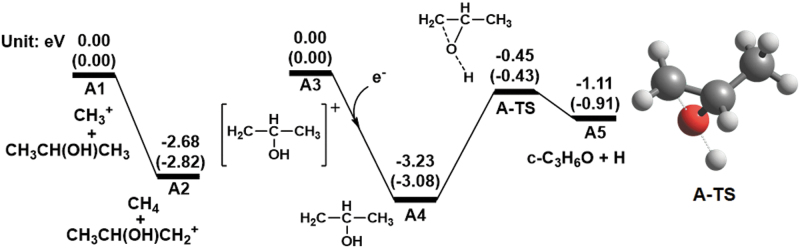
Computed energy diagrams for path **A** at the CCSD(T)/aug-cc-pVTZ level of theory together with the optimized structure of the transition state at the B3LYP/aug-cc-pVTZ level of theory. The values in parentheses represent the energies calculated at the B3LYP/aug-cc-pVTZ level of theory. Relative energies with respect to **A1** and **A3** are expressed in electron volts. The energy of **A3** was calculated for the state with an electron added to the structure of CH_3_CH(OH)CH_2_^+^.

Path **A** consists of two reactions: (1) C_3_H_7_O^+^ and CH_4_ generation from CH_3_^+^ and CH_3_CH(OH)CH_3_ (A-1), and (2) the formation of *c*-C_3_H_6_O (A-2). Figure 1 shows the computed energy diagrams for path **A** at the CCSD(T)/aug-cc-pVTZ level of theory. Although CH_3_CH(OH)CH_3_ has several stable rotamers, their relative energies of a few kcal mol^−1^ (Kahn and Bruice, 2005; Snow *et al.,* 2011) are sufficiently low to not affect the entire computed energy diagram. Therefore, we only focused on the specific conformation that proceeds the desired reactions, in addition to Paths **B** and **C**. CH_3_^+^ and CH_3_CH(OH)CH_3_ (**A1**) are initially transformed into CH_4_ and CH_3_CH(OH)CH_2_^+^ (**A2**), respectively, through hydrogen abstraction. The reaction is exothermic (2.68 eV) without any reaction barriers, indicating that the reaction proceeds spontaneously when CH_3_^+^ and CH_3_CH(OH)CH_3_ come into contact with each other. Through reduction (*i.e.,* the addition of an electron (e^−^)), CH_3_CH(OH)CH_2_^+^ produces *c*-C_3_H_6_O and H (**A5**); the complex of CH_3_CH(OH)CH_2_^+^ and e^−^ (**A3**) transforms into CH_3_CH(OH)CH_2_ (**A4**) and leads to **A5** via a transition state (**A-TS**). Because **A-TS** and **A5** have negative energies relative to **A3**, *c*-C_3_H_6_O formation can occur by adding an e^−^ to CH_3_CH(OH)CH_2_^+^. This suggests that path **A** is a *c*-C_3_H_6_O formation pathway.

#### Path **B**

**FIG. 2.**
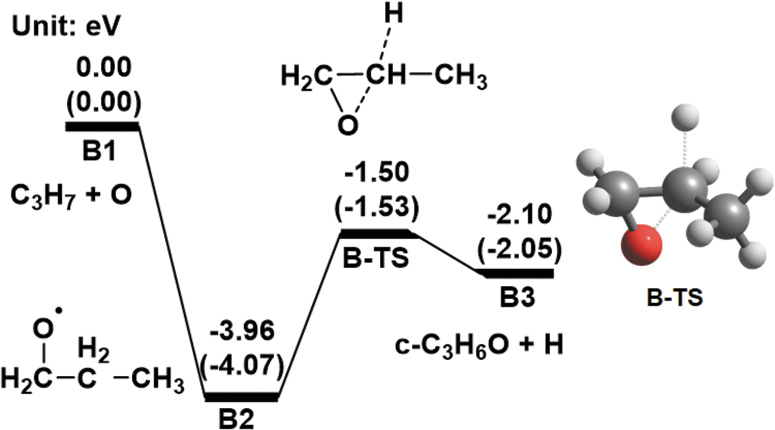
Computed energy diagrams for path **B** at the CCSD(T)/aug-cc-pVTZ level of theory together with the optimized structure of the transition state at the B3LYP/aug-cc-pVTZ level of theory. The values in parentheses represent the energies calculated at the B3LYP/aug-cc-pVTZ level of theory. Relative energies with respect to **B1** are expressed in electron volts.

We considered path **B** for the formation of *c*-C_3_H_6_O and H from C_3_H_7_^•^ and O (triplet). Our calculations reveal that the addition of C_3_H_7_^•^ and O (triplet) (**B1**) produces CH_2_OCH_2_CH_3_ (**B2**) with an energy of −3.96 eV without any reaction barriers, as shown in Fig. 2. **B2** produces *c*-C_3_H_6_O and H (**B3**) via a transition state (**B-TS**). Because **B-TS** and **B3** are calculated to have negative energies relative to **B1**, *c*-C_3_H_6_O formation can occur by the addition of C_3_H_7_^•^ and O (triplet). This indicates that path **B** is also a *c*-C_3_H_6_O formation pathway, similar to path **A**.

#### Path **C**

**FIG. 3.**
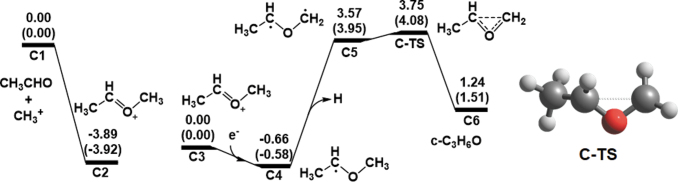
Computed energy diagrams for path **C** at the CCSD(T)/aug-cc-pVTZ level of theory together with the optimized structure of the transition state at the B3LYP/aug-cc-pVTZ level of theory. The values in parentheses represent the energies calculated at the B3LYP/aug-cc-pVTZ level of theory. Relative energies with respect to **C1** and **C3** are expressed in electron volts.

Finally, we considered path **C**, which comprises two reactions, as follows: (1) CH_3_CHOCH_3_^+^ generation from CH_3_^+^ and CH_3_CHO (C-1), and (2) the formation of *c*-C_3_H_6_O (C-2). Figure 3 shows the computed energy diagrams for path **C**. The addition of CH_3_^+^ and CH_3_CHO (**C1**) initially produces CH_3_CHOCH_3_^+^ (**C2**). The reaction is exothermic (-3.89 eV) without any reaction barriers, which indicates that the reaction proceeds automatically when CH_3_^+^ and CH_3_CHO come into contact with each other. CH_3_CHOCH_3_^+^ then forms CH_3_CHOCH_3_^•^ (**C4**) by the addition of an e^−^ with an energy of -0.66 eV. CH_3_CHOCH_3_^•^ generates CH_3_CH^•^OCH_2_^•^ (**C5**) with hydrogen cleavage, and the reaction is endothermic (4.22 eV). Finally, *c*-C_3_H_6_O is produced by the C–C bond formation of CH_3_CH^•^OCH_2_^•^ via a transition state (**C-TS**). Because the energies for **C5** and **C-TS** are remarkably higher than those for **C3**, we do not consider path **C** as a formation pathway of *c*-C_3_H_6_O.

### 3.2. Photo-absorption property of CPL

We investigated the photo-absorption property of the chiral species for Lyα with an emission of 10.2 eV (121.6 nm) and discussed the possibility of photolysis through the absorption of CPL in the Lyα region.

In previous works, the CD spectrum of *c*-C_3_H_6_O was measured in the vacuum ultraviolet region up to 8.2 eV (151 nm) (Carnell *et al.,* 1991) and 9.0 eV (138 nm) (Breest *et al.,* 1994). In the spectrum, three band maxima appeared at 7.1 eV (175 nm), 7.7 eV (161 nm), and 8.4 eV (148 nm), and the CD peak sign at 7.7 eV was opposite to the other peak signs at 7.1 and 8.4 eV (Breest *et al.,* 1994). The simulated CD spectrum was also obtained from DFT and post-HF calculations (Carnell *et al.,* 1991; Turner and Apponi, 2001; Miyahara *et al.,* 2009; Varsano *et al.,* 2009; Kröner, 2015). The results were qualitatively in agreement with the experimental CD spectrum. However, even the SAC-CI calculations, which provided a good theoretical description of the first two spectral bands, overestimated the rotatory strengths for the state corresponding to the third band (Miyahara *et al.,* 2009; Kröner, 2015). According to the benchmark studies on CD simulations using TD-DFT calculations (Turner and Apponi, 2001; Jang *et al.,* 2018), the M06-2X, CAM-B3LYP, and ωB97X-D functionals are feasible (Jang *et al.,* 2018). Moreover, rotatory strengths require doubly augmented basis sets of at least triple zeta quality to reach a similar degree of convergence with the CAM-B3LYP functional (Turner and Apponi, 2001). The electronic excitation energies, oscillator strengths, and rotational strengths of *c*-C_3_H_6_O and CH_3_CH(OH)CH_2_^+^ (**A3** in Fig. 1), which are chiral species obtained under the *c*-C_3_H_6_O formation pathways, were calculated from the TD-DFT calculations with the CAM-B3LYP functional and daug-cc-pVQZ basis set.

**FIG. 4.**
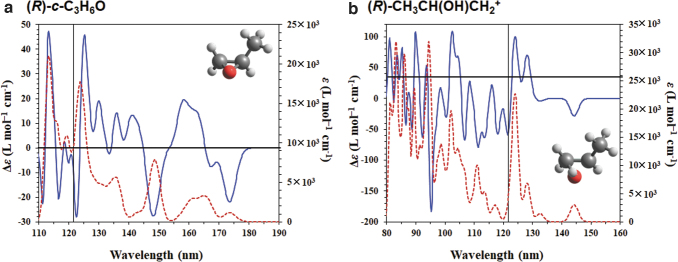
Simulated CD (blue line) and UV (red dashed line) spectra of (**a**) (*R*)-*c*-C_3_H_6_O and (**b**) (*R*)-CH_3_CH(OH)CH_2_^+^ using CAM-B3LYP/daug-cc-pVQZ level of theory. The spectra were obtained using Gaussian functions with a broadening parameter of 0.1 eV. The black line represents the position at 121.1 nm.

**FIG. 5.**
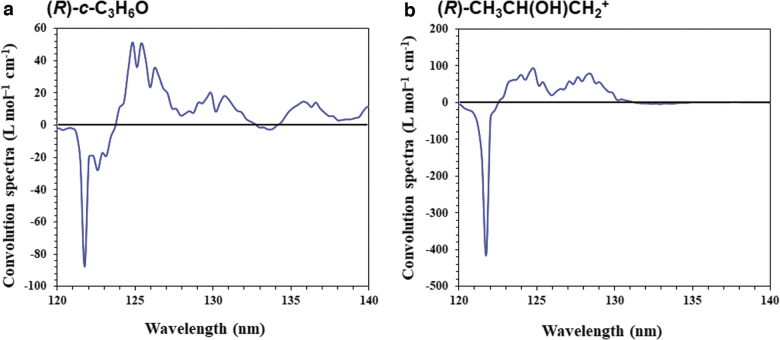
Convolution of the observational LAE spectrum and calculated CD spectra of (**a**) (*R*)-*c*-C_3_H_6_O and (**b**) (*R*)-CH_3_CH(OH)CH_2_^+^ between 120 and 140 nm.

Figure 4a shows the computed CD and UV spectra of (*R*)-*c*-C_3_H_6_O. Three main peaks between 145 and 180 nm, with one positive and two negative signs, are observed and are consistent with the experimental observations (Breest *et al.,* 1994). Herein, we focus on the photoabsorption property of the LAE spectrum with an intense peak at 10.2 eV (121.6 nm), although we cannot compare these results with the experimental results around the higher energy region because of the lack of experimental observations in this region. The calculated UV spectrum shows that *c*-C_3_H_6_O absorbs light at approximately 120 nm because of its strong oscillator strength. The calculated CD spectrum has a slightly negative sign at 121.6 nm. Figure 4b also shows the computed CD and UV spectra of (*R*)-CH_3_CH(OH)CH_2_^+^. We consider the convolution of our CD spectra and the LAE spectrum (Shibuya *et al.,* 2014), as shown in Fig. 5. In both cases, intense peaks near 121.6 nm are observed. Therefore, we suggest that both *c*-C_3_H_6_O and CH_3_CH(OH)CH_2_^+^ can absorb the Lyα line.

A previous experimental study demonstrated that irradiating *c*-C_3_H_6_O in the gas phase at 185 nm generated propanal and acetone (Paulson *et al.,* 1977). The experimental results and our computational results of the CD spectrum imply the occurrence of photolysis of *c*-C_3_H_6_O by Lyα, although the wavelength is different from that used in the previous experimental measurements. Determining the detailed photolysis reaction mechanism is a future challenge; however, the present results provide a possibility of the formation of *c*-C_3_H_6_O homochirality in space. Experimental verification is required to examine the photolysis by CPL irradiation in detail.

## 4. Conclusions

In this study, we investigated the formation and possible photolysis of *c*-C_3_H_6_O, a chiral molecule first detected in the Sagittarius B2 star-forming region (McGuire *et al.,* 2016), under interstellar conditions. We focused on the previously proposed *c*-C_2_H_4_O formation mechanism (Dickens *et al.,* 1997; Turner and Apponi, 2001; Bennett *et al.,* 2005) to investigate the reaction pathways of *c*-C_3_H_6_O formation at the atomic level, which cannot be detected by space observation due to the short lifetime of most of the intermediates. The computed energy diagrams show two energetically downhill pathways for *c*-C_3_H_6_O formation. One pathway consisted of two reactions: (1) C_3_H_7_O^+^ and CH_4_ generation from CH_3_^+^ and CH_3_CH(OH)CH_3_, and (2) *c*-C_3_H_6_O formation from C_3_H_7_O^+^ and e^−^. The other pathway produced *c*-C_3_H_6_O from C_3_H_7_^•^ and O (triplet). Therefore, *c-*C_3_H_6_O was produced by CH_3_^+^ and CH_3_CH(OH)CH_3_ or C_3_H_7_^•^ and O (triplet), as indicated by our quantum chemical calculations. Because the reactants, the product, and several intermediates with a long lifetime can be detected by observation, the pathway proposed by our calculations is plausible in an interstellar environment. Furthermore, the CD spectra obtained from DFT and post-HF calculations indicate that *c*-C_3_H_6_O and CH_3_CH(OH)CH_2_^+^ could absorb UV-CPL from the LAE spectrum. This suggests that the photolysis of *c*-C_3_H_6_O or CH_3_CH(OH)CH_2_^+^ under CPL irradiation could induce chiral symmetry breakage. Infrared CPL was detected within the high-mass star-forming regions of OMC-1 (Bailey *et al.,* 1998), and the infrared CP image also shows that the infrared CPL region was spatially extended around young stellar objects (Fukue *et al.,* 2010; Kwon *et al.,* 2013, 2014, 2016, 2018). In addition, LAEs were observed in the early phase of the galactic evolution (Shibuya *et al.,* 2014). These experiments and measurements, in addition to our present calculation results, are consistent with the astrophysical scenario of the origin of homochirality. To prove this scenario, further experimental verification is required to examine the photolysis by CPL irradiation in detail. The short-lived intermediates discovered in our calculations, the formation energies of various reactions, and other calculated spectroscopic data are expected to be beneficial for their experimental verification.

## Supplementary Material

Supplemental data

Supplemental data

Supplemental data

## Abbreviations Used

CD: circular dichroism
DFT: density functional theory
*ee*: enantiomeric excess
LAEs: Lyman-α emitters
Lyα: Lyman-α
post-HF: post-Hartree-Fock
(TD)-DFT: time-dependent density functional theory
UV-CPL: ultraviolet circularly polarized light

## Notes

Associate Editor: Christopher McKay
