# Supplementary material for: Theoretical Investigation into a Possibility of Formation of Propylene Oxide Homochirality in Space
Source: Astrobiology. 2022 Oct 31;22(11):1330–6. doi: 10.1089/ast.2022.0005 (PMC9618371; doi:10.1089/ast.2022.0005)
Supplement: Supplemental data [file Suppl_TableS1.pdf]

Table S1. Energetically lowest excited state energies E (eV), corresponding wavelengths λ (nm), oscillator strengths f^{osc}, rotatory strengths R (10^{-40} esu^2 cm^2) of (R)-c-C_3H_6O.

E	λ	f^{osc}	R
7.15	173.53	0.0086	-14.9551
7.45	166.39	0.016	-7.4579
7.56	163.97	0.0134	8.4957
7.72	160.71	0.0174	6.4593
7.86	157.68	0.006	10.2697
8.29	149.63	0.0129	-5.0921
8.32	149.03	0.0104	-5.043
8.34	148.73	0.0277	0.2222
8.41	147.5	0.0043	-4.1727
8.44	146.93	0.006	-8.6095
8.44	146.84	0.0067	0.6831
8.48	146.23	0.0025	1.5287
8.53	145.37	0.0013	-0.9032
8.58	144.52	0.0017	1.6092
8.66	143.13	0.0014	-0.325
8.68	142.88	0.0034	3.2848
8.72	142.14	0.0027	1.1081
8.83	140.41	0.0015	6.0399
8.98	138.01	0.0032	-2.3134
9.04	137.18	0.0034	2.0423
9.07	136.64	0.0016	1.1069
9.09	136.36	0.0186	-2.0936
9.11	136.05	0.0034	1.8767
9.16	135.4	0.0096	7.4576
9.17	135.15	0.0009	1.3733
9.22	134.56	0.0021	-0.1076
9.25	134.05	0.0012	-0.6089
9.26	133.85	0.0182	-7.6179
9.31	133.21	0.0026	2.8178

E	λ	f^{osc}	R
9.32	133.08	0.0008	0.9097
9.34	132.8	0.0031	-0.2605
9.40	131.86	0.0046	-1.3616
9.43	131.46	0.0009	-0.3332
9.44	131.4	0.0055	5.2229
9.45	131.16	0.0032	1.7686
9.46	131.09	0.0097	-2.1139
9.47	130.88	0.0005	-2.1092
9.52	130.26	0.0025	-0.7429
9.56	129.66	0.0008	1.4243
9.58	129.47	0.0112	19.9733
9.61	129.05	0.0089	-12.5893
9.70	127.83	0.0051	-1.6725
9.71	127.66	0.0147	0.7079
9.73	127.39	0.006	-2.2026
9.77	126.94	0.0052	8.8459
9.78	126.74	0.0004	-0.5726
9.83	126.1	0.0004	-0.3525
9.86	125.81	0.0119	0.1597
9.87	125.62	0.0111	-11.3471
9.90	125.29	0.0175	27.0492
9.93	124.93	0.0069	1.5643
9.95	124.67	0.0213	2.0915
9.98	124.28	0.0053	-2.9918
10.01	123.86	0.0556	10.5426
10.09	122.86	0.0561	-20.3495
10.11	122.69	0.0014	0.6791
10.14	122.34	0.0007	-0.145
10.17	121.89	0.0056	0.9487
10.18	121.82	0.0042	-3.0982
10.19	121.67	0.0014	-1.4474
10.20	121.53	0.0049	1.6203
10.24	121.05	0.0023	-0.5265

E	λ	f^{osc}	R
10.26	120.83	0.0104	12.8211
10.28	120.66	0.0092	-5.4139
10.31	120.32	0.0039	2.5503
10.33	119.99	0.0053	-1.891
10.35	119.85	0.0047	-7.1815
10.39	119.39	0.0337	-10.0813
10.41	119.08	0.0004	1.2976
10.43	118.88	0.0117	15.0249
10.46	118.51	0.0108	8.1776
10.48	118.31	0.0034	-9.5918
10.49	118.16	0.0065	-6.6411
10.52	117.84	0.0119	3.9804
10.56	117.45	0.0035	2.6115
10.59	117.12	0.0013	-0.5391
10.60	117.03	0.0017	-2.1457
10.62	116.73	0.0021	-2.2025
10.65	116.38	0.0019	-1.1983
10.66	116.27	0.0304	2.5336
10.67	116.16	0.0281	-11.3893
10.70	115.9	0.0053	-1.1179
10.76	115.2	0.0022	-0.3042
10.78	115.06	0.0258	14.9444
10.81	114.73	0.0004	-0.2795
10.83	114.52	0.0029	-6.3353
10.87	114.11	0.0289	8.2429
10.89	113.88	0.0092	1.0715
10.92	113.57	0.0181	-9.2751
10.92	113.54	0.0121	-4.7754
10.94	113.33	0.0145	7.7469
10.95	113.29	0.0138	0.2512
10.96	113.09	0.0206	8.0251
10.98	112.93	0.0132	17.7247
10.98	112.91	0.0129	0.1533

E	λ	f^{osc}	R
11.01	112.61	0.0043	6.3384
11.01	112.58	0.0191	-6.5118
11.08	111.96	0.0053	2.2234
11.09	111.82	0.0137	-13.7215
11.10	111.69	0.0091	-4.5479
