# Supplementary material for: Theoretical Investigation into a Possibility of Formation of Propylene Oxide Homochirality in Space
Source: Astrobiology. 2022 Oct 31;22(11):1330–6. doi: 10.1089/ast.2022.0005 (PMC9618371; doi:10.1089/ast.2022.0005)
Supplement: Supplemental data [file Suppl_TableS2.pdf]

Table S2. Energetically lowest excited state energies *E* (eV), corresponding wavelengths *λ* (nm), oscillator strengths f^{osc}, rotatory strengths *R* (10^{-40} esu^2 cm^2) of (*R*)-$CH_3CH(OH)CH_2^+$.

E	*λ*	f^{osc}	*R*
8.60	144.2	0.0223	-15.962
9.35	132.55	0.0109	-2.1893
9.68	128.1	0.0509	35.4607
10.00	124.02	0.1596	41.3713
10.10	122.82	0.0132	27.4139
10.17	121.96	0.0151	-47.0678
10.53	117.78	0.0135	-32.8376
10.64	116.5	0.0141	18.8721
10.91	113.61	0.0294	-26.2635
10.93	113.43	0.009	-1.3054
11.15	111.22	0.0492	-36.9723
11.21	110.59	0.0285	4.3955
11.36	109.11	0.0202	-5.0921
11.46	108.16	0.0041	21.0249
11.61	106.85	0.0455	-31.3585
11.72	105.79	0.039	-4.5044
11.84	104.75	0.0389	22.8489
11.91	104.09	0.0517	6.3521
12.03	103.05	0.0357	15.189
12.15	102.02	0.0484	90.49
12.17	101.91	0.0821	-56.6264
12.35	100.42	0.0324	-17.7346
12.40	100.03	0.0177	6.7774
12.50	99.17	0.035	-6.6972
12.58	98.53	0.0486	19.4922
12.60	98.38	0.0207	-10.5382
12.72	97.48	0.0187	12.9737
12.77	97.12	0.023	-9.719
12.77	97.07	0.0264	-13.6731

E	λ	f^{osc}	R
12.95	95.73	0.012	-13.1502
13.04	95.1	0.0721	-51.3418
13.05	95.04	0.0266	-10.7678
13.11	94.59	0.0307	-2.5083
13.15	94.28	0.1175	4.9028
13.21	93.89	0.0409	15.3144
13.30	93.26	0.0451	15.2059
13.35	92.9	0.0333	3.513
13.36	92.83	0.0288	-14.2258
13.45	92.17	0.0242	-20.3169
13.51	91.79	0.0251	1.8881
13.54	91.56	0.0059	-5.1343
13.61	91.12	0.0336	-3.1958
13.62	91.01	0.0412	18.7284
13.79	89.9	0.0103	23.9531
13.84	89.62	0.0631	3.375
13.88	89.33	0.0772	32.5971
13.90	89.22	0.0142	13.7834
13.92	89.06	0.014	-28.8645
13.94	88.96	0.0096	-31.714
14.01	88.52	0.0023	-2.0973
14.05	88.27	0.0066	12.735
14.13	87.75	0.0193	-14.6777
14.16	87.59	0.0658	8.5342
14.19	87.4	0.0314	8.5972
14.19	87.37	0.0184	-2.3064
14.26	86.97	0.0083	3.503
14.31	86.67	0.0012	-6.3755
14.33	86.51	0.0185	-2.8469
14.36	86.34	0.0598	-14.6651
14.38	86.23	0.0083	11.5585
14.40	86.11	0.059	-18.9463
14.44	85.87	0.0423	-33.5193

E	λ	f^{osc}	R
14.45	85.79	0.0373	64.6967
14.53	85.34	0.0276	2.0274
14.54	85.31	0.0039	-8.1008
14.56	85.15	0.0017	1.0836
14.57	85.08	0.0005	-3.8319
14.60	84.91	0.0271	28.2667
14.68	84.48	0.036	14.0758
14.69	84.42	0.0155	-27.7089
14.72	84.23	0.0086	2.6181
14.73	84.16	0.0024	-0.4456
14.77	83.93	0.0125	-1.7191
14.80	83.78	0.01	0.475
14.81	83.72	0.0237	17.6441
14.85	83.51	0.0422	4.4931
14.88	83.31	0.0726	8.1532
14.91	83.15	0.011	-2.3067
14.93	83.06	0.0196	-1.3275
14.94	83.01	0.0255	3.0179
14.95	82.92	0.0349	-5.7664
15.00	82.69	0.0036	3.8149
15.01	82.61	0.0317	15.5332
15.04	82.44	0.0181	-24.84
15.07	82.27	0.011	7.4537
15.09	82.17	0.0281	-12.1814
15.13	81.95	0.0135	2.7882
15.13	81.94	0.0047	9.3202
15.17	81.74	0.0091	-3.2998
15.18	81.67	0.0191	13.9462
15.21	81.55	0.0011	-0.4837
15.22	81.45	0.007	4.6352
15.24	81.35	0.0334	-9.863
15.28	81.16	0.0299	9.5318
15.29	81.12	0.0159	1.9997

E	λ	f^{osc}	R
15.31	80.98	0.0239	-0.9459
15.34	80.86	0.0038	8.7873
15.35	80.78	0.0288	14.9861
15.36	80.75	0.0047	4.3889
15.38	80.61	0.0072	-5.6518
