# Supplementary material for: Theoretical Investigation into a Possibility of Formation of Propylene Oxide Homochirality in Space
Source: Astrobiology. 2022 Oct 31;22(11):1330–6. doi: 10.1089/ast.2022.0005 (PMC9618371; doi:10.1089/ast.2022.0005)
Supplement: Supplemental data [file Suppl_TableS3.pdf]

Table S3. Calculated dissymmetry factor ($g_{abs} = \Delta\varepsilon/\varepsilon$) obtained from Fig. 4 at several wavelengths for (a) *c*-C_3H_6O and (b) $CH_3CH(OH)CH_2^+$.

(a) *c*-C_3H_6O				
Wavelength (nm)	121.1	148.1	158.3	173.4
g_{abs} ($\times 10^3$)	−0.424	−3.66	11.6	−18.8
(b) $CH_3CH(OH)CH_2^+$				
Wavelength (nm)	121.1	124.0	128.1	144.2
g_{abs} ($\times 10^3$)	−23.5	4.41	10.2	−9.34
